# Comparing domain- and intensity-specific physical activity in coronary heart disease and non-CHD individuals

**DOI:** 10.1038/s41598-024-52764-3

**Published:** 2024-02-01

**Authors:** Seon Young Goo, Mi Kyung Lee, Dong Hoon Lee, Dong - Hyuk Park, Tae Ho Lee, Chan Joo Lee, Jong-Young Lee, Justin Y. Jeon

**Affiliations:** 1 Department of Sport Industry Studies and Exercise Medicine Center for Diabetes and Cancer Patients, Yonsei University, 50, Yonsei-Ro, Seodaemun-Gu, Seoul, Republic of Korea; 2 Frontier Research Institute of Convergence Sports Science, Yonsei University, Seoul, Republic of Korea; 3 Department of Nutrition, Harvard T.H. Chan School of Public Health, Boston, MA USA; 4 Division of Cardiology, Department of Internal Medicine, Severance Hospital, Yonsei University College of Medicine, Seoul, Republic of Korea; 5 Division of Cardiology, Department of Internal Medicine, Kangbuk Samsung Hospital, School of Medicine, Sungkyunkwan University, 29, Saemunan-Ro, Jongno-Gu, Seoul, Republic of Korea; 6 Exercise Medicine Center for Diabetes and Cancer Patients, ICONS, Yonsei University, Seoul, Republic of Korea; 7 Cancer Prevention Center, Yonsei Cancer Center, Yonsei University College of Medicine, Seoul, Republic of Korea

**Keywords:** Cardiology, Diseases, Health care

## Abstract

Although increase in physical activity is important to improve prognosis of cardiac patients in addition to hospital-based exercise cardiac rehabilitation, their physical activity levels are not properly understood. This study aimed to examine domain- and intensity-specific physical activity in individuals with coronary heart disease (CHD) and compare them with non-CHD individuals. Data from the Korean National Health and Nutrition Examination Survey (KNHANES) from 2014 to 2019 were analyzed, including 1083 CHD patients and 38,532 non-CHD individuals. The inclusion criteria were age 19 years or older and data not missing for CHD information. Before and after propensity score matching (PSM) for age, sex, body mass index, education, household income, alcohol intake, and smoking status, domain (leisure, work, transportation)—and intensity (moderate, vigorous) -specific physical activity participation levels were compared between individuals with and without CHD. Before PSM, CHD individuals were older, less educated, more sedentary, and participated less in PAs compared to non-CHD individuals. After PSM, CHD individuals had similar levels of domain-specific PAs. However, they had higher work-related PA levels (29.7 ± 209.6 vs. 42.1 ± 291.3 min/week *p* = 0.022) and more sedentary time (487.2 ± 224.2 vs. 514.1 ± 228.7. *p* = 0.003) than those without CHD. Subgroup analysis revealed lower leisure-related PA in men with CHD (63.5 ± 165.5 vs. 47.3 ± 140.2, *p* < 0.05) and higher work-related PA in women with CHD (18.9 ± 159.7 vs. 57.1 ± 397.5, *p* < 0.01). Among those < 65 years of age, individuals with CHD spent more time sedentary than individuals without CHD. CHD individuals are not physically inactive compared with non-CHD individuals who are similar in sociodemographic status and lifestyle. CHD patients’ PA levels may have been underestimated.

## Introduction

Coronary heart diseases (CHD) are a leading cause of global mortality and a significant contributor to disability^1^. In the Republic of Korea, the number of people hospitalized for heart disease in 2018 increased four-fold from 2002 and was the second leading cause of death in 2019^2^. The most prevalent heart disease is CHD which includes angina and myocardial infarction (MI). Preventing recurrent CHD is important considering the increasing number of risk factors given the aging population in Korea^2^.

Exercise-based cardiac rehabilitation (CR) is a class 1 recommendation for preventing recurrent CHD events^3^. Exercise improves physical health and reduces the risk of MI, all-cause hospitalization, all-cause mortality, and cardiovascular mortality in individuals with CHD^4^. In developing effective physical activity program for patients with CHD, it is important to understand physical activity participation levels. While studies have reported physical activity behaviors of individuals with various CVDs, they were either baseline physical activity levels from intervention studies or prospective cohort studies. It is challenging to compare the physical activity levels of individuals with CHD to individuals without CHD. In addition, studies have suggested different domains of physical activity may have unique health effects^7^. Leisure activities (e.g., sports and exercise) are performed at the discretion of an individual and are not essential for daily living. Work activities (e.g., construction, manufacturing) include all activities within occupational settings, and transportation activities (e.g., walking, cycling) include activities done as a mode of transportation. There is limited research on domain-specific physical activity among individuals with CHD^8–10^. Any potential differences between individuals (with and without CHD) matched on sociodemographic and lifestyle factors are unknown. The CR team should consider any unique physical activity patterns or characteristics of individuals with CHD when planning and implementing CR programming.

In South Korea, the Korean National Health and Nutrition Examination Survey (KNHNES) is an annually conducted nationwide survey (including domain-specific physical activity). This survey aims to recruit a representative population-based sample and includes individuals with CHD and other chronic diseases. The objective of our study was to determine domain-specific physical activity behaviors, intensities, and sedentary time among individuals with CHD. The secondary objective of our study was to explore differences in these activity-related variables by comparing individuals with CHD to a sample of individuals without CHD matched for socioeconomic status and lifestyle factors. We hypothesized that individuals with CHD would report doing less physical activity in all domains than individuals without CHD.

## Methods

### Participants and data source

We used the 2014–2019 KNHANES data. The KNHANES is conducted annually by the Korea Centers for Disease Control and Prevention (KCDC). This nationwide cross-sectional survey recruits approximately 10,000 non-institutionalized civilians each year through a stratified multistage probability sampling method. KNHANES data allows for monitoring of public health and health trends and policy planning and development. KNHANES includes health interviews, health examinations, and nutrition surveys. The KNHANES data resource profiles^11^ provides further details on the KNHANES study procedures.

**Figure 1:**
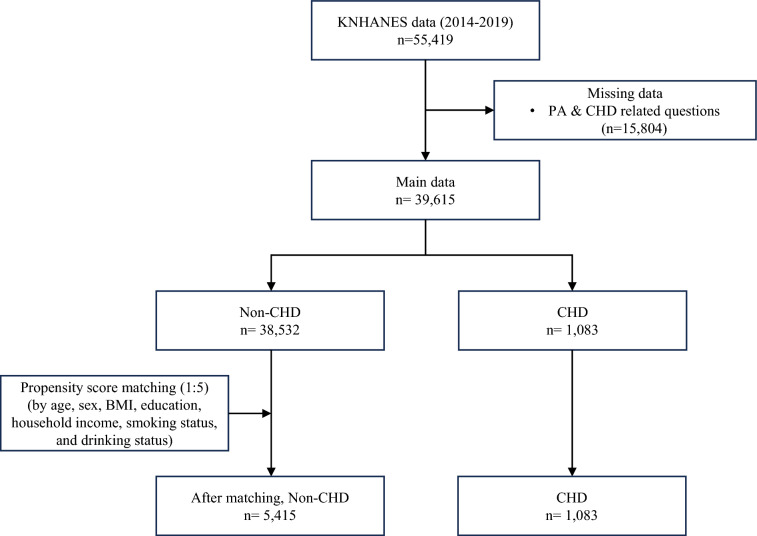
Flowchart of the study. KNHANES: Korean national health and nutrition examination survey, CHD: coronary heart disease, BMI: body mass index, PA: physical activity.

From the 2014–2019 KNHANES data, we included all participants over 18 years old (N = 55,419). We excluded participants with missing values for CHD-related questions and physical activity (N = 15,804). After the exclusion, we identified 38,532 individuals without CHD and 1083 individuals with CHD (Fig. 1). Individuals without CHD were those who reported that they had never been diagnosed with any CHD. Individuals with CHD were those who reported that they had been previously diagnosed with angina or MI. Individuals with both angina and MI were categorized in the MI group, as MI is a more severe condition that can occur if angina worsens. Individuals were asked if they had a) a history of being diagnosed by a doctor, b) currently suffering from the disease, or c) currently receiving treatment for the disease. We counted those who answered *yes* to at least one of the items as individuals with angina or MI.

### Measures

In KNHANES, demographic, socioeconomic, and physical activity information were collected via self-reported questionnaires and face-to-face interviews using standardized questionnaires. Data were collected in a mobile inspection vehicle. All participants provided written informed consent. Angina and MI were the only types of CHD included in the KNHANES data.

Sociodemographic and descriptive variables included age, sex, body mass index (BMI) (kg/m^2^), educational attainment, marital status, employment status, household income, smoking status, alcohol intake, and age at CHD diagnosis. BMI was taken from the measured weight and height to the nearest 0.1 kg and 0.1 cm and was calculated as the ratio of weight in kilograms to height in squared meters. Household income was calculated by dividing the monthly household income by the square root of the household size and grouped into quartiles (i.e., low, moderate-low, moderate-high, and high), education attainment (i.e., ≤ elementary, middle school, high school, ≥ college), smoking status (i.e., current smoker, past smoker, never smoker), and alcohol intake (i.e., < 1/month, 2–4/month, ≥ 2/week). We calculated the time from CHD diagnosis by subtracting the age at the first CHD diagnosis from the age at the time of completing the survey.

Physical activity was measured using the Global Physical Activity Questionnaire (GPAQ)^12^. The GPAQ captures physical activity domain (i.e., work, leisure, transport) and intensity (i.e., vigorous, moderate), frequency, and duration. Vigorous intensity activity was defined as any activity that required a considerable amount of physical effort and caused heavy breathing and a high heart rate. Moderate intensity activity was defined as any activity that required less physical effort and slightly increased breathing and heart rate. Work and leisure physical activity included information on intensity, but not physical activity for transport. Total physical activity was calculated by summing the time/duration spent in each activity domain. The validity and reliability of the GPAQ in Korean population have been previously described^13^.

### Statistical analysis

“SPSS version 25 (IBM Corp., Armonk, NY, USA) was used for all analyses. For propensity score matching (PSM), we used SAS 15.1 (SAS Institute Inc., Cary, North Carolina, USA). Propensity score, which indicates the probability that an individual being assigned to a particular condition (CHD in the current study), was calculated using logistic regression based on predefined factors including age, sex, BMI, education, household income, smoking status, and alcohol intake that are recognized as major risk factors for both physical activity and CHD. Using these calculated propensity scores, individuals with CHD (cases) and without CHD (controls) were matched in a 1:5 ratio using the 1:N case-control matching macro with greedy algorithms provided the SAS group^14^. The number of matches per subject was selected as 1:5 to increase the precision of estimates and statistical power.”

For descriptive analysis, continuous variables including physical activity were expressed as means (standard deviation), and categorical variables were presented as percentages. To test the normality of the data, the Kolmogorov–Smirnov test was used. To compared the differences between individuals with and without CHD, Chi-squared and t-tests were used for normally distributed variables. When the data were not normally distributed, Kruskal Wallis and the Mann–Whitney U tests were used to compare variables between the groups. Statistical significance was set at *p* < 0.05.

### Ethics approval

The Institutional Review Board of the Korea Centers for Disease Control and Prevention approved KNHANES study protocols (2013-12EXP-03-5C, 2018–01-03-P-A, 2018–01-03-C-A). All parts of the study were carried out in agreement with the Declaration of Helsinki.

## Results

We identified 38,532 non-CHD individuals and 1083 CHD patients in the KNHANES data from 2014 to 2019. Before matching, the mean age of individuals without CHD was 51 ± 16.8 years and 68.9 ± 8.9 years for individuals with CHD. BMI among individuals without CHD was 23.9 ± 3.6 and 24.8 ± 3.2 kg/m^2^ for individuals with CHD. After matching, the mean age for both samples was 69 years and the mean BMI was 24.7 kg/m^2^. Table 1 shows significant differences across all variables when comparing individuals with and without CHD before PSM. After PSM, a total of 5415 non-CHD individuals were matched and differences for only employment status (*p* < 0.05) and smoking status (*p* < 0.05) remained. Among individuals with CHD, 61.5% (n = 666) reported angina and 38.5% (n = 417) reported MI. There were no missing data for variables in Table 1.

**Table 1:** Characteristics of individuals with and without CHD before and after propensity score matching.

Variables	Before matching			After matching*		
	Non-CHD	CHD	*p* value	Non-CHD	CHD	*p* value
Number of participants	(N = 38,532)	(N = 1083)		(N = 5415)	(N = 1083)	
Age (years)	51 ± 16.8	68.9 ± 8.9	** < 0.001**	69.1 ± 9.3	68.9 ± 8.9	0.35
Sex (M/F)	16,545/21,987	689/497	** < 0.001**	3005/2410	689/497	0.15
BMI (kg/m^2^)	23.9 ± 3.6	24.8 ± 3.2	** < 0.001**	24.7 ± 3.4	24.8 ± 3.2	0.25
Education						
≤ Elementary	7713 (20.1)	529 (45.4)	** < 0.001**	2574 (48)	529 (45.4)	0.12
Middle school	3810 (9.9)	188 (16.1)		774 (14.4)	188 (16.1)	
High school	12,642 (33)	284 (24.4)		1179 (22)	284 (24.4)	
≥ College	14,164 (37)	165 (14.2)		831 (15.5)	165 (14.2)	
Marital status						
Married	31,926 (82.9)	1161 (97.9)	** < 0.001**	5319 (98.2)	1161 (97.9)	0.56
Single	6604 (17.1)	25 (2.1)		96 (1.8)	25 (2.1)	
Employment status						
Employed	23,286 (60.7)	466 (39.7)	**0.005**	2332 (43.5)	466 (39.7)	**0.04**
Unemployed	15,064 (39.3)	707 (60.3)		3029 (56.5)	707 (60.3)	
Household income						
Low	7178 (18.7)	459 (38.7)	**0.005**	2124 (39.4)	459 (38.7)	0.38
Low-mid	9502 (24.7)	319 (26.9)		1393 (25.9)	319 (26.9)	
Mid-high	10,412 (27.1)	240 (20.2)		997 (18.5)	240 (20.2)	
High	11,301 (29.4)	168 (14.2)		874 (16.2)	168 (14.2)	
Smoking status						
Current	6864 (17.9)	188 (16)	**0.005**	869 (16.2)	188 (16)	**0.004**
Past	8114 (21.2)	446 (38.1)		1763 (32.9)	446 (38.1)	
Never	23,311 (60.9)	538 (45.9)		2731 (50.9)	538 (45.9)	
Drinking status						
Low (< 1/month)	13,299 (39.1)	473 (49.6)	**0.006**	1903 (44.6)	473 (49.6)	0.07
Moderate (2 ~ 4/month)	12,435 (36.6)	241 (25.3)		1191 (27.9)	241 (25.3)	
High (≥ 4/week)	8287 (24.4)	240 (25.2)		1172 (27.5)	240 (25.2)	
Angina		666 (61.5)			666 (61.5)	
Myocardial infarction		417 (38.5)			417 (38.5)	

Values are mean ± SD, N (%), *Propensity score matching (1:5), non-CHD individuals matched by age, sex, BMI, education, household income, smoking status, and drinking status, BMI (body mass index), M (male), F (female).

Significant values are in bold.

Table 2 shows physical activity intensity and minutes per week for different domains between individuals with and without CHD. Before PSM, individuals with CHD reported significantly fewer minutes of vigorous physical activity, leisure physical activity, physical activity for transport, and total physical activity compared to individuals without CHD. Individuals with CHD reported significantly more sedentary time than those without CHD. After PSM, we found no significant differences in leisure time and transport-related physical activity between groups. Individuals with CHD spent significantly more time in work-related physical activity and significantly more time sedentary compared to those without CHD.

**Table 2:** Physical activity participation of individuals with and without CHD before and after propensity score matching.

Variables	Before matching					After matching*				
	Non-CHD (N = 38532)	Missing	CHD (N = 1186)	Missing	*p*	Non-CHD (N = 5415)	Missing	CHD (N = 1083)	Missing	*p*
Physical activity intensity										
Vigorous (min/week)	26.1 ± 129.3	11,647	10.8 ± 72.0	381	** < 0.001**	15 ± 124.7	1270	10.8 ± 72.0	278	0.341
Moderate (min/week)	82.5 ± 255.6	11,651	69.7 ± 305.2	383	0.166	60 ± 208.0	1270	69.7 ± 305.2	280	0.645
Physical activity domain										
Work (min/week)	48.7 ± 267.1	11,638	42.1 ± 291.3	384	0.495	29.7 ± 209.6	1270	42.1 ± 291.3	281	**0.022**
Leisure time (min/week)	59.4 ± 139.9	152	36.4 ± 115.3	10	** < 0.001**	44.5 ± 136.6	41	36.4 ± 115.3	10	0.250
Transportation (min/week)	114.8 ± 191.0	217	95.6 ± 173.0	13	** < 0.001**	103 ± 193.1	79	95.6 ± 173.0	13	0.203
Total (min/week)	227.3 ± 380.2	11,721	176.9 ± 382.1	800	** < 0.001**	180.8 ± 338.3	1308	176.9 ± 382.1	283	0.264
Sedentary time (min/day)	485.6 ± 217.0	674	514.1 ± 228.7	46	** < 0.001**	487.2 ± 224.2	245	514.1 ± 228.7	42	**0.003**
Total walking time (min/week)	247.7 ± 355.0	235	233.5 ± 335.1	17	0.155	250.2 ± 398.8	72	233.5 ± 335.1	17	0.623

Values are mean ± SD.

*Propensity score matching (1:5).

Total physical activity = (minutes of work physical activity per week) + (minutes of leisure time physical activity per week) + (minutes of transportation physical activity per week).

Significant values are in bold.

Table 3 shows subgroup analyses of domain-specific physical activity according to sex (men vs. women) and age (< 65 years vs. ≥ 65 years). Leisure-time physical activity was significantly lower in men with CHD, and work-related physical activity was higher in women with CHD. Both men and women with CHD reported significantly more sedentary time then those without CHD. Among those < 65 years of age, individuals with CHD spent significantly more time sedentary than individuals without CHD.

**Table 3:** Physical activity levels of individuals with and without CHD by sex and age after PSM.

	Work PA	%*	Leisure PA	%	Transportation PA	%	Total PA	%	Sedentary time
Men									
Non-CHD (n = 3005)	38.3 ± 241.7	5.1	63.5 ± 165.5	24.4	114.1 ± 219.5	49.4	222.2 ± 386.6	48.2	467.9 ± 218.5
CHD (n = 627)	30.4 ± 166.0	5.6	47.3 ± 140.2^†^	19.9	99.2 ± 187.0	46.1	185.5 ± 296.3	42.6	505.2 ± 230.0^†^
Women									
Non-CHD (n = 2410)	18.9 ± 159.7	3.7	20.6 ± 81.8	10.2	88.9 ± 152.5	49	128.3 ± 255.3	41.9	512.6 ± 229.1
CHD (n = 456)	57.1 ± 397.5^†^	6.4	22.8 ± 74.9	12.5	90.9 ± 150.6	50	165.9 ± 470.2	42.6	517.7 ± 229.1^†^
< 65 years									
Non-CHD (n = 1559)	44.0 ± 257.9	6.6	66.7 ± 146.7	29.3	114.2 ± 234.7	50.4	231.2 ± 413.9	51.2	440.5 ± 209.3
CHD (n = 311)	72.5 ± 354.3	8.7	52.5 ± 131.7	27.4	99.5 ± 172.3	48.3	233 ± 451.5	47.6	478.3 ± 242.1^†^
≥ 65 years									
Non-CHD (n = 3856)	23.8 ± 185.8	3.6	35.4 ± 131.2	13.5	98.3 ± 173.1	48.9	159.9 ± 298.9	43.1	507.2 ± 227.4
CHD (n = 772)	29.7 ± 260.6	4.8	30.7 ± 111.3	12.5	94.1 ± 172.9	47.5	158.9 ± 348.4	40.5	523.5 ± 223.1

Values are mean ± SD minutes per week or %

PA (physical activity).

CHD = angina + myocardial infarction.

*The proportion of participants in each subgroup engaging in a physical activity domain.

^†^*p*-value of < 0.05.

There were no significant associations between any physical activity variables (i.e., intensity and domain) and time since angina or MI diagnosis (see Supplementary Table s1). Individuals who reported work-related physical activity had significantly fewer sedentary time min/day and higher vigorous, moderate, leisure, and total physical activity than those who did not report work-related physical activity (see Supplementary Table s2). Physical activity guideline (more than 150 min per week of moderate intensity physical activity) satisfaction rates of participants with and without CHD were 11.3%, 6.4% and 15.7%, 5.4%, for male and female respectively (see Supplementary Table s3).

## Discussion

We hypothesized individuals with CHD would be less physically active than the general population who do not have CHD. Before PSM, we observed a significantly lower amount of time spent in vigorous, leisure, transport, and total physical activity participation and significantly more sedentary time among individuals with CHD than those without CHD. Considering the differences in sociodemographic and lifestyle variables between the two groups, we used PSM to compare the groups. After matching, individuals with CHD had equivalent physical activity levels to individuals without CHD other than work-related physical activity. Leisure physical activity time was significantly lower in men with CHD, while women reported higher work-related physical activity. Among those < 65 years of age, individuals with CHD spent significantly more time sedentary than those without CHD.

We observed similar physical activity levels in individuals with and without CHD when matched for sociodemographic and lifestyle variables. One study in the United States reported the age-standardized percentage of adults with and without CHD who participated in physical activity at recommended levels. Their data suggested individuals with CHD participated in significantly less total (40% meeting recommendations vs. 49% meeting recommendations), moderate (32% vs. 37%), and vigorous (22% vs. 29%) physical activity compared with individuals without CHD^15^. Another study reported physical activity participation rates of coronary patients from European countries (moderate or vigorous physical activity for at least 20 min once or more times a week: 38%) but not in comparison to those without CHD^16^. Furthermore, discrepancies exist between the findings of the current study and those of Baker et al.^17^, which reported a significant reduction in objectively measured physical activity compared to the health control group. One of the primary distinctions between our study and Baker et al.'s study is the average age of participants. Notably, participants in Baker et al. (2019) are significantly older (no disease: male 61.0 ± 8.0 and female 60.7 ± 7.7 years old; chronic disease: male 65.5 ± 7.1, female 63.5 ± 7.5). Considering that vigorous physical activity tends to decrease with age, the observed lower physical activity participation in individuals with CVD compared to those without CVD could be partially attributed to the higher age among individuals with CVD. In our study, we employed propensity score matching. Consequently, we were able to compare physical activity participation levels between participants who were matched for age, BMI, education, household income, alcohol intake, and smoking status. This approach enhances the robustness of our comparisons, minimizing potential confounding effects and providing a more accurate assessment of the impact of chronic disease on physical activity participation.

When interpreting our data, one should note that physical activity levels are generally low among Korean adults in comparison to other countries. A recent study on physical activity prevalence among Korean adults between the ages of 60 and 69 years reported that 10.2% were engaging in high levels of physical activity^18^, which is lower than older Czech adults (24.9–28.3%)^19^ and Iranians between the ages of 55 and 64 years (23.7%)^20^. In addition, some people with CHD may have recognized the importance of PA and tried to be more physically active. Stewart and colleagues reported that 34% of individuals with CHD increased their physical activity after their CHD diagnosis^21^ and similar results have been reported in European samples^16^. The extent to which people are motivated to increase the amount of activity after diagnosis and the effect over time is unclear and may vary by country and/or ethnicity.

Physical activity domains (i.e., leisure, work, and transportation) may have varying associations with CHD. Leisure activity has an inverse linear association with CVD risk factors and mortality^22,23^. Associations of other physical activity domains with cardiovascular health remain less clear. Regarding work-related physical activity, research has suggested shift work may increase the risk of CVD events, and work-related physical activity may increase all-cause mortality in men^24,25^. As for transportation-related physical activity, adults living in walkable neighborhoods had lower 10-year CVD risk compared to those who do not live in walkable neighborhoods^26^. Referred to as the *physical activity paradox*, it is unclear why occupational or work-related physical activity does not confer similar health benefits as leisure or recreational physical activities^7^. It is noteworthy that there was no discernible difference in work-related physical activity before PSM; however, individuals with CHD exhibited higher levels of work-related physical activity. This observation may be attributed to significant disparities in age (51 ± 16.8 vs. 68.9 ± 8.9 years old), sex, BMI (23.9 ± 3.6 vs. 24.8 ± 3.2, kg/m^2^), marital status, and employment status before matching. Prior to PSM, individuals without CHD were notably younger, more likely to be single, leaner, and employed. Consequently, the comparison of work-related physical activity after PSM matching enhances the validity of the analysis by accounting for these baseline differences and ensuring a more balanced and unbiased assessment between the groups.

In our subgroup analyses, we observed a lower engagement in leisure physical activity among men with CHD, while women with CHD demonstrated higher levels of work-related physical activity. It is noteworthy that there was no significant difference in work-related physical activity before matching, but a notable disparity emerged after matching. Upon closer examination through subgroup analyses, we found that this divergence in work-related physical activity was specific to women. The reason for the heightened work-related physical activity in women with CHD remains unclear, especially considering a smaller proportion of participants with CHD were employed. It is essential to recognize that the GPAQ not only assesses occupational physical activity but also encompasses physical activity related to unpaid work, household chores, and harvesting food/crops. Additionally, it is pertinent to highlight that the types of employment were comparable between individuals with and without CHD. Therefore, the observed differences in work-related physical activity in our study may be attributed, at least in part, to variances in socioeconomic status. It is crucial to further investigate and understand the factors contributing to the observed patterns in physical activity among women with CHD in various domains. Among men and women who were < 65 years of age and had CHD, significantly more time was spent sedentary than those without CHD.

It is noteworthy that individuals with CHD showed significantly higher sedentary time in our study, in agreement with findings from previous studies^27,28^. Since replacing sedentary time with physical activity may reduce inflammation^29^, CVD mortality, and all-cause mortality^30^, individuals with CHD should be encouraged to limit the amount of time spent in sedentary pursuits. Replacing sedentary time with leisure physical activity should be strongly recommended. Sociodemographic characteristics of individuals with CHD who report high work-related physical activity levels should be further analyzed. Whether individuals with CHD who report high work-related physical activity levels need additional leisure activity should be a question for future research in this area.

Shorter sedentary time and higher levels of leisure physical activity are associated with beneficial effects on CVD^22,30^. Higher and prolonged sedentary time has adverse effects on CVD markers, such as blood pressure, high-density lipoprotein, low-density lipoprotein, and C-reactive protein^31^. Similar to previous research, individuals with CHD were spending significantly more time sedentary compared to those without CHD. Since all activities performed in a single day are interdependent, longer sedentary time could mean less time spent in physical activity. Recent studies point to the need to incorporate more sophisticated measures such as posture-discriminating accelerometry and distinguish between sedentary patterns. Future studies should also apply isotemporal substitution modeling approaches to determine the impact (on health outcomes) of reducing sedentary time and increasing time spent in other physical activity domains.

One limitation of our study is that the diagnosis of CHD was based on a subjective questionnaire. This method may lead to instances where cases of angina, unrelated to CHD, are included, and some patients may not have experienced a MI. Another limitation stems from the broad definition of CHD, encompassing a spectrum of severity, ranging from well-controlled angina with minimal symptoms to those experiencing worsening conditions. Moreover, our study's measurement of physical activity relied on subjective, self-reported data. Individuals with CHD in our study might have provided socially desirable responses to the physical activity questionnaires, potentially leading to an overreporting of their physical activity levels. Although objective/device-based methods, such as accelerometers, cannot distinguish between different domains of physical activity, our findings remain credible. For future research, a more comprehensive approach would involve simultaneously implementing both objective and self-report methods for assessing physical activity. This would provide a more nuanced and accurate understanding of individuals' activity levels and contribute to a more robust interpretation of the relationship between CHD and physical activity. Moreover, although we adjusted for major demographic and lifestyle related factors, we cannot rule out unmeasured confounding by other diseases and factors that may influence physical activity level and CHD. Despite these limitations, our study stands as the first to compare physical activity levels between individuals with and without CHD after meticulous matching on sociodemographic and lifestyle variables on a national scale.

## Conclusions

In conclusion, individuals with CHD) exhibit comparable levels of physical activity to their non-CHD counterparts with similar sociodemographic status and lifestyle, although in our study, both CHD and non-CHD are not very physically active. Interestingly, a notable difference emerges as CHD individuals demonstrate higher levels of work-related physical activity and spend more time in sedentary pursuits compared to those without CHD. It is established that reducing sedentary time and engaging in more leisure physical activity yield positive effects on recurrent CVD. Therefore, it is advisable to recommend the substitution of sedentary time with leisure physical activity for individuals with CHD, promoting cardiovascular health and overall well-being.

### Supplementary Information

Supplementary Information.

## Data Availability

The datasets generated during and/or analysed during the current study are available from the corresponding author on reasonable request.

## Abbreviations

CHD: Coronary heart disease
KNHANES: Korean National Health and Nutrition Examination Survey
PSM: Propensity score matching
CR: Cardiac rehabilitation
MI: Myocardial infarction
BMI: Body mass index
GPAQ: Global physical activity questionnaire
CVD: Cardiovascular disease
