# Supplementary material for: Comparing domain- and intensity-specific physical activity in coronary heart disease and non-CHD individuals
Source: Sci Rep. 2024 Feb 1;14:2622. doi: 10.1038/s41598-024-52764-3 (PMC10831062; doi:10.1038/s41598-024-52764-3)
Supplement: Supplementary file 1 — Supplementary Information. [file 41598_2024_52764_MOESM1_ESM.docx]

**Supplementary Tables**

Table 1. Physical activity intensity and domains according to the time from CHD diagnosis

|  |  |  | | |  | |  | |  |  | |  | |  | |  |
| --- | --- | --- | --- | --- | --- | --- | --- | --- | --- | --- | --- | --- | --- | --- | --- | --- |
| Variables | N (%) | Vigorous | Moderate | Work | | Leisure | | Transportation | | | Total activity | | Walking time | | Sedentary time | |
| **Angina (years)** | 644 |  |  |  | |  | |  | | |  | |  | |  | |
| < 3 | 162 (25.2) | 18.3±102.1 | 130.9±469.2 | 87.5±442.6 | | 51.2±158.3 | | 129.3±245.8 | | | 286.2±589.9 | | 233.2±268.8 | | 527.6±244.2 | |
| 3 ~ 6 | 146 (22.7) | 5.1±27.4 | 46±145.8 | 26.2±136.8 | | 28.5±72.8 | | 106.4±194 | | | 162.7±252.7 | | 273.8±432.6 | | 497.3±224.2 | |
| 6 ~ 12 | 176 (27.3) | 11.1±59.7 | 73.7±207.3 | 32.3±186.4 | | 51.3±135.1 | | 86.1±132.8 | | | 170.2±268.1 | | 269±444.7 | | 491.7±216.5 | |
| > 12 | 160 (24.8) | 8.1±39.5 | 106.9±528.1 | 75.7±520.5 | | 34.5±102.2 | | 123.8±187.8 | | | 231.5±593.2 | | 221.3±252.6 | | 522±219.9 | |
| p value |  | .892 | .353 | .743 | | .275 | | .483 | | | .876 | | .914 | | .393 | |
| **MI (years)** | 368 |  |  |  | |  | |  | | |  | |  | |  | |
| < 3 | 100 (27.1) | 7.0±50.1 | 22.8±62.5 | 16.9±85.5 | | 16.5±44.4 | | 98.7±184.7 | | | 148.1±222.3 | | 246.1±313 | | 500±222 | |
| 3 ~ 6 | 87 (23.6) | 29.7±158.3 | 145.5±700.1 | 142.1±710.5 | | 34.8±136.5 | | 84.2±135.8 | | | 270.7±767 | | 243.7±387.9 | | 448.2±211.8 | |
| 6 ~ 12 | 99 (26.8) | 7.3±39.7 | 54.8±154.7 | 7.3±38.2 | | 50.9±153.5 | | 81.3±133.3 | | | 139.7±205.7 | | 200.3±255.1 | | 503.5±245.5 | |
| > 12 | 82 (22.5) | 5.28±38.5 | 84.9±265.4 | 58.9±253.3 | | 54±123.5 | | 137.2±219.7 | | | 195.8±398.2 | | 277.9±331.9 | | 501.4±240 | |
| p value |  | .229 | .717 | .217 | | .437 | | .287 | | | .838 | | .456 | | .323 | |

Values are mean ± SD minutes per week, Total physical activity = (minutes of work physical activity per week) + (minutes of leisure time physical activity per week) + (minutes of transportation physical activity per week)

Table 2. Physical activity intensity and domain according to work-related physical activity participation

|  |  |  |  | |  | |  |  | |  | |  |  |  |
| --- | --- | --- | --- | --- | --- | --- | --- | --- | --- | --- | --- | --- | --- | --- |
| Variables | N (%) | Vigorous | | Moderate | | Leisure | | | Transportation | | Total | | Walking time | Sedentary time |
| **Work physical activity participation** | 802 (100) |  | |  | |  | | |  | |  | |  |  |
| No | 738 (92) | 8.1±63.1 | | 29.5±101 | | 37.6±128.3 | | | 95.2±174.9 | | 132.9±218.5 | | 230.3±368.9 | 506.1±228.8 |
| Yes | 64 (8) | 43.1±135.8 | | 534.6±909.8 | | 50±102.9 | | | 107.1±177.8 | | 692.6±1010.7 | | 146.3±154.7 | 403.1±187.1 |
| p value |  | **< .001** | | **< .001** | | **0.002** | | | .234 | | **< .001** | | .550 | **.001** |

Values are mean ± SD minutes per week, Total physical activity = (minutes of work physical activity per week) + (minutes of leisure time physical activity per week) + (minutes of transportation physical activity per week).

Table 3. Physical activity guideline satisfaction rates of participants with and without CHD

|  |  |  | Inactive  (0 METs mins/week) | Insufficiently active  (< 600 METs mins/week) | Active  (600 ≥ METs mins/week) |
| --- | --- | --- | --- | --- | --- |
| Leisure | CHD | Male (N = 622) | 497 (79.9) | 55 (8.8) | 70 (11.3) |
|  |  | Female (N = 451) | 394 (87.4) | 28 (6.2) | 29 (6.4) |
|  | Non-CHD | Male (N = 2,987) | 2,255 (75.5) | 263 (8.8) | 469 (15.7) |
|  |  | Female (N = 2,387) | 2,141 (89.7) | 117 (4.9) | 129 (5.4) |
| Leisure +  Transportation | CHD | Male (N = 622) | 274 (44.1) | 150 (24.1) | 198 (31.8) |
|  |  | Female (N = 448) | 201 (44.9) | 123 (27.5) | 124 (27.7) |
|  | Non-CHD | Male (N = 2,973) | 1,193 (40.1) | 635 (21.4) | 1,145 (38.5) |
|  |  | Female (N = 2,362) | 1,089 (46.1) | 647 (27.4) | 626 (26.5) |

Physical activity guideline: moderate intensity 30 minutes at least 5 days/week (AHA ACC guidelines for secondary prevention for patients with coronary and other atherosclerotic vascular disease 2006 update). 30 minutes * 5 days * 4 METs = 600 METs mins/week, Vigorous physical activity = 8 METs, Moderate/transportation physical activity = 4 METs
